# Supplementary material for: Growth State-Dependent Expression of Arachidonate Lipoxygenases in the Human Endothelial Cell Line EA.hy926
Source: Cells. 2022 Aug 10;11(16):2478. doi: 10.3390/cells11162478 (PMC9406857; doi:10.3390/cells11162478)
Supplement: Supplementary file 1 [file cells-11-02478-s001.zip › cells-1811009-supplementary.pdf]

SUPPLEMENTARY FIGURES and LEGENDS

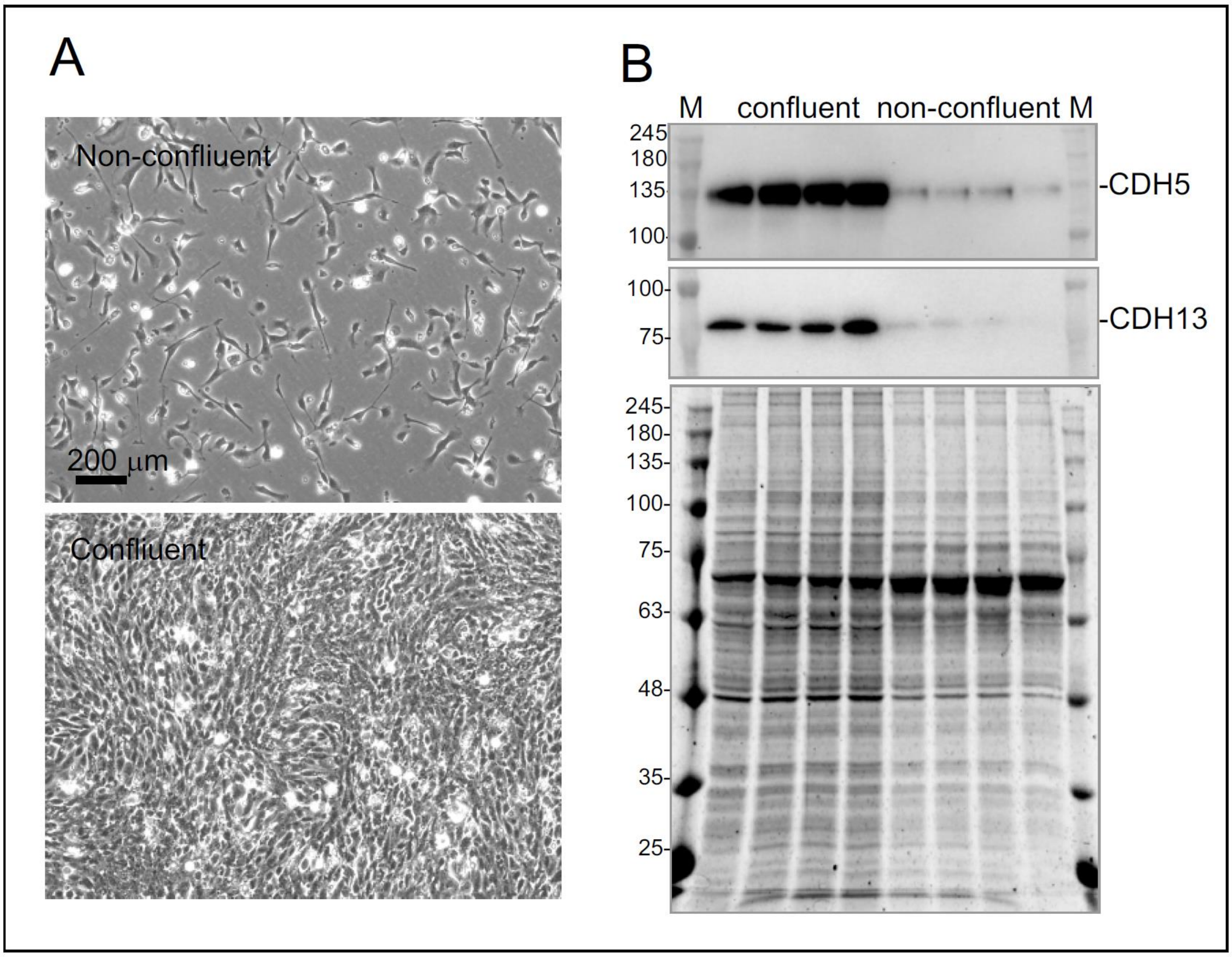

Figure S1. Marker proteins of confluent and non-confluent EA.hy926 cells. (A): Bright field microscopic images showing non-confluent and confluent EA.hy926 cells. (B): Immunoblots of lysates prepared in quadruplicate showing levels of CDH5 and CDH13 in confluent and non-confluent EA.hy926 endothelial cells as well as the respective Oriole-stained gel showing total protein loading. M: molecular mass markers.

ALOX15-201: ENSP00000293761

```
  1 AGAAGGAGCGAAACATCTTTGAGCAAGATGGGTCTCTACCGCATCCGCGTGTCCACTGGG   60
    ...........................ATGGGTCTCTACCGCATCCGCGTGTCCACTGGG   33
    ...........................-M--G--L--Y--R--I--R--V--S--T--G-   11

 61 GCCTCGCTCTATGCCGGTTCCAACAACCAGGTGCAGCTGTGGCTGGTCGGCCAGCACGGG  120
 34 GCCTCGCTCTATGCCGGTTCCAACAACCAGGTGCAGCTGTGGCTGGTCGGCCAGCACGGG   93
 12 -A--S--L--Y--A--G--S--N--N--Q--V--Q--L--W--L--V--G--Q--H--G-   31

121 GAGGCGGCGCTCGGGAAGCGACTGTGGCCCGCACGGGGCAAGGAGACAGAACTCAAGGTG  180
 94 GAGGCGGCGCTCGGGAAGCGACTGTGGCCCGCACGGGGCAAGGAGACAGAACTCAAGGTG  153
 32 -E--A--A--L--G--K--R--L--W--P--A--R--G--K--E--T--E--L--K--V-   51

181 GAAGTACCGGAGTATCTGGGGCCGCTGCTGTTTGTGAAACTGCGCAAACGGCACCTCCTT  240
154 GAAGTACCGGAGTATCTGGGGCCGCTGCTGTTTGTGAAACTGCGCAAACGGCACCTCCTT  213
 52 -E--V--P--E--Y--L--G--P--L--L--F--V--K--L--R--K--R--H--L--L-   71

241 AAGGACGACGCCTGGTTCTGCAACTGGATCTCTGTGCAGGGCCCCGGAGCCGGGGACGAG  300
214 AAGGACGACGCCTGGTTCTGCAACTGGATCTCTGTGCAGGGCCCCGGAGCCGGGGACGAG  273
 72 -K--D--D--A--W--F--C--N--W--I--S--V--Q--G--P--G--A--G--D--E-   91

301 GTCAGGTTCCCTTGTTACCGCTGGGTGGAGGGCAACGGCGTCCTGAGCCTGCCTGAAGGC  360
274 GTCAGGTTCCCTTGTTACCGCTGGGTGGAGGGCAACGGCGTCCTGAGCCTGCCTGAAGGC  333
 92 -V--R--F--P--C--Y--R--W--V--E--G--N--G--V--L--S--L--P--E--G-  111

361 ACCGGCCGCACTGTGGGCGAGGACCCTCAGGGCCTGTTCCAGAAACACCGGGAAGAAGAG  420
334 ACCGGCCGCACTGTGGGCGAGGACCCTCAGGGCCTGTTCCAGAAACACCGGGAAGAAGAG  393
112 -T--G--R--T--V--G--E--D--P--Q--G--L--F--Q--K--H--R--E--E--E-  131

421 CTGGAAGAGAGAAGGAAGTTGTACCGGTGGGGAAACTGGAAGGACGGGTTAATTCTGAAT  480
394 CTGGAAGAGAGAAGGAAGTTGTACCGGTGGGGAAACTGGAAGGACGGGTTAATTCTGAAT  453
132 -L--E--E--R--R--K--L--Y--R--W--G--N--W--K--D--G--L--I--L--N-  151

481 ATGGCTGGGGCCAAACTATATGACCTCCCTGTGGATGAGCGATTTCTGGAAGACAAGAGA  540
454 ATGGCTGGGGCCAAACTATATGACCTCCCTGTGGATGAGCGATTTCTGGAAGACAAGAGA  513
152 -M--A--G--A--K--L--Y--D--L--P--V--D--E--R--F--L--E--D--K--R-  171

541 GTTGACTTTGAGGTTTCGCTGGCCAAGGGGCTGGCCGACCTCGCTATCAAAGACTCTCTA  600
514 GTTGACTTTGAGGTTTCGCTGGCCAAGGGGCTGGCCGACCTCGCTATCAAAGACTCTCTA  573
172 -V--D--F--E--V--S--L--A--K--G--L--A--D--L--A--I--K--D--S--L-  191
```

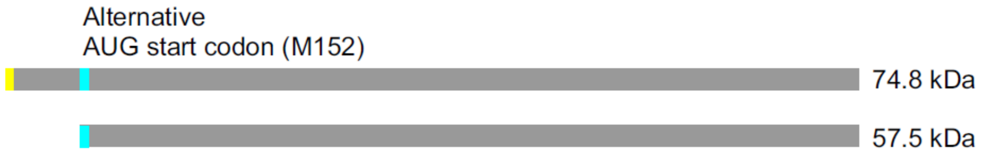

Figure S2. Alternative AUG start codon in *ALOX15* gene. Nucleotide sequence of the exons (blue letters), coding sequence (codons: black letters), and protein sequence (magenta letters) of *ALOX15*. Beneath the sequence is a representation showing the relative theoretical molecular weight of ALOX15 based on the consensus start codon (yellow highlight) compared to the alternative start codon (cyan highlight).

ALOX15B-201: ENSP00000369520

```
  1 GTCCACTGGGCTTGGAGTCAGTGGCAATAACCAGGGGCAATAACCAGGCGTGTCCCAGGG    60
    ............................................................
    ............................................................

 61 GGGAGCCCCGCTCTGCAGCCCTGTGCGCCGTAGAGAGCTGGACTTAGGCTGGCAGCATGG   120
    ........................................................ATGG     4
    ........................................................-M--     1

121 CCGAGTTCAGGGTCAGGGTGTCCACCGGAGAAGCCTTCGGGGCTGGCACATGGGACAAAG   180
  5 CCGAGTTCAGGGTCAGGGTGTCCACCGGAGAAGCCTTCGGGGCTGGCACATGGGACAAAG    64
  2 A--E--F--R--V--R--V--S--T--G--E--A--F--G--A--G--T--W--D--K--    21

181 TGTCTGTCAGCATCGTGGGGACCCGGGGAGAGAGCCCCCCACTGCCCCTGGACAATCTCG   240
 65 TGTCTGTCAGCATCGTGGGGACCCGGGGAGAGAGCCCCCCACTGCCCCTGGACAATCTCG   124
 22 V--S--V--S--I--V--G--T--R--G--E--S--P--P--L--P--L--D--N--L--    41

241 GCAAGGAGTTCACTGCGGGCGCTGAGGAGGACTTCCAGGTGACGCTCCCGGAGGACGTAG   300
125 GCAAGGAGTTCACTGCGGGCGCTGAGGAGGACTTCCAGGTGACGCTCCCGGAGGACGTAG   184
 42 G--K--E--F--T--A--G--A--E--E--D--F--Q--V--T--L--P--E--D--V--    61

301 GCCGAGTGCTGCTGCTGCGCGTGCACAAGGCGCCCCCAGTGCTGCCCCTGCTGGGGCCCC   360
185 GCCGAGTGCTGCTGCTGCGCGTGCACAAGGCGCCCCCAGTGCTGCCCCTGCTGGGGCCCC   244
 62 G--R--V--L--L--L--R--V--H--K--A--P--P--V--L--P--L--L--G--P--    81

361 TGGCCCCGGATGCCTGGTTCTGCCGCTGGTTCCAGCTGACACCGCCGCGGGGCGGCCACC   420
245 TGGCCCCGGATGCCTGGTTCTGCCGCTGGTTCCAGCTGACACCGCCGCGGGGCGGCCACC   304
 82 L--A--P--D--A--W--F--C--R--W--F--Q--L--T--P--P--R--G--G--H--   101

421 TCCTCTTCCCCTGCTACCAGTGGCTGGAGGGGGCGGGGACCCTGGTGCTGCAGGAGGGTA   480
305 TCCTCTTCCCCTGCTACCAGTGGCTGGAGGGGGCGGGGACCCTGGTGCTGCAGGAGGGTA   364
102 L--L--F--P--C--Y--Q--W--L--E--G--A--G--T--L--V--L--Q--E--G--   121

481 CAGCCAAGGTGTCCTGGGCAGACCACCACCCTGTGCTCCAGCAACAGCGCCAGGAGGAGC   540
365 CAGCCAAGGTGTCCTGGGCAGACCACCACCCTGTGCTCCAGCAACAGCGCCAGGAGGAGC   424
122 T--A--K--V--S--W--A--D--H--H--P--V--L--Q--Q--Q--R--Q--E--E--   141

541 TTCAGGCCCGGCAGGAGATGTACCAGTGGAAGGCTTACAACCCAGGTTGGCTCACTGCC    600
425 TTCAGGCCCGGCAGGAGATGTACCAGTGGAAGGCTTACAACCCAGGTTGGCTCACTGCC    484
142 L--Q--A--R--Q--E--M--Y--Q--W--K--A--Y--N--P--G--W--P--H--C--   161

601 TGGATGAAAAGACAGTGGAAGACTTGGAGCTCAATATCAAATACTCCACAGCCAAGAATG   660
485 TGGATGAAAAGACAGTGGAAGACTTGGAGCTCAATATCAAATACTCCACAGCCAAGAATG   544
162 L--D--E--K--T--V--E--D--L--E--L--N--I--K--Y--S--T--A--K--N--   181

661 CCAACTTTTATCTACAGGCTGGCTCTGCTTTTGCAGAGATGAAAATCAAGGGGTTGCTGG   720
545 CCAACTTTTATCTACAGGCTGGCTCTGCTTTTGCAGAGATGAAAATCAAGGGGTTGCTGG   604
182 A--N--F--Y--L--Q--A--G--S--A--F--A--E--M--K--I--K--G--L--L--   201

721 ACCGCAAGGGGCTCTGGAGGAGTCTGAATGAGATGAAAAGGATCTTCAACTTCCGGAGGA   780
605 ACCGCAAGGGGCTCTGGAGGAGTCTGAATGAGATGAAAAGGATCTTCAACTTCCGGAGGA   664
202 D--R--K--G--L--W--R--S--L--N--E--M--K--R--I--F--N--F--R--R--   221
```

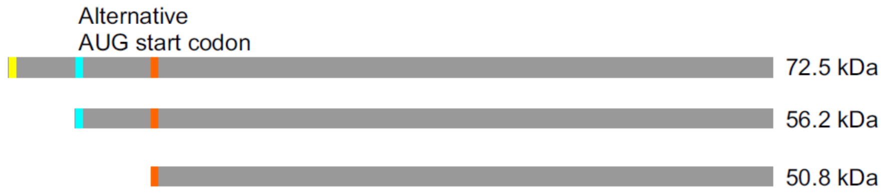

Figure S3. Alternative AUG start codons in *ALOX15B* gene. Nucleotide sequence of the exons (blue letters), coding sequence (codons: black letters), and protein sequence (magenta letters) of *ALOX15B*. Beneath the sequence is a representation showing the relative theoretical molecular weight of ALOX15B based on the consensus start codon (yellow highlight) compared to the alternative first and second start codon (cyan and red highlights, respectively).

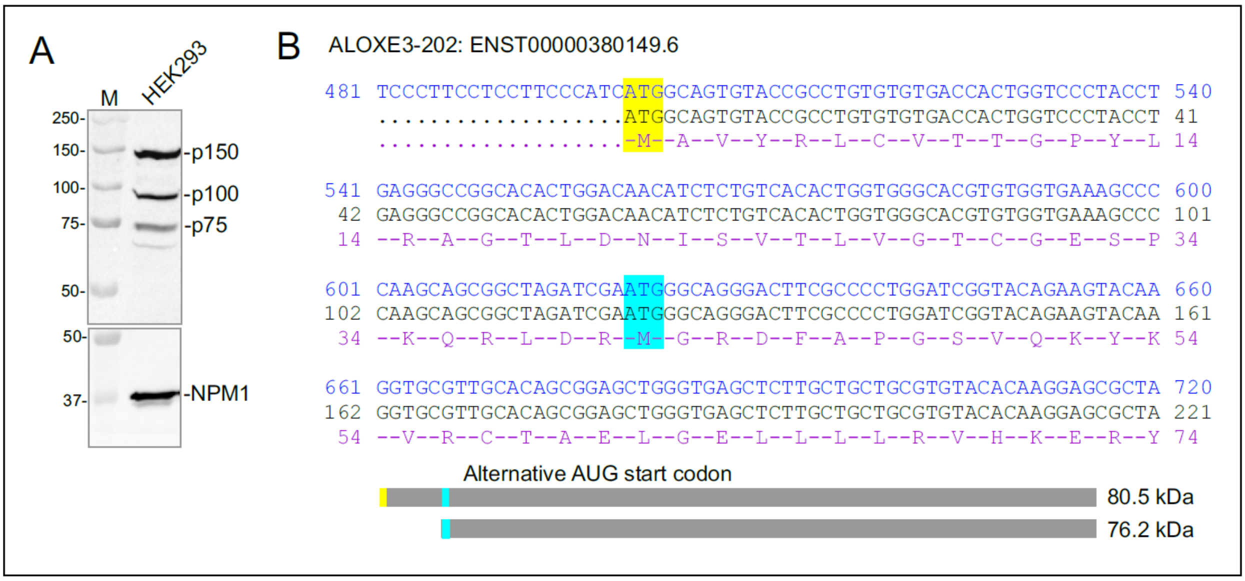

Figure S4. Immunoblotting for ALOXE3 in HEK293 cells and alternative AUG start codon in *ALOXE3* gene. (A): Lysates of HEK293 cells were prepared and immunoblotted for ALOXE3 and NPM1 (loading control). A representative blot is depicted. M: molecular mass markers. (B): Nucleotide sequence of the exons (blue letters), coding sequence (codons: black letters), and protein sequence (magenta letters) of *ALOXE3*. Beneath the sequence is a representation showing the relative theoretical molecular weight of ALOXE3 based on the consensus start codon (yellow highlight) compared to the alternative start codon (cyan highlight).
